# Overvaluation of weight and shape in obesity: a comparative study between people with and without binge eating disorder

**DOI:** 10.3389/fpsyg.2024.1414455

**Published:** 2024-06-24

**Authors:** Neli Escandón-Nagel, Maribel Peró-Cebollero, Antoni Grau, José Soriano, Guillem Feixas

**Affiliations:** ^1^Departament of Psychology, Facultad de Ciencias de la Salud, Universidad Católica de Temuco, Temuco, Chile; ^2^Departament de Psicología Social i Psicología Quantitativa, Facultat de Psicología, Institut de Neurociències de la Universitat de Barcelona, Universitat de Barcelona Institute of Complex Systems, Universitat de Barcelona, Barcelona, Spain; ^3^ITA Salud Mental, Clariane, Spain; ^4^Hospital de la Santa Creu i Sant Pau, Barcelona, Spain; ^5^Department of Clinical Psychology and Psychobiology, Institut de Neurociències, Universitat de Barcelona, Barcelona, Spain

**Keywords:** obesity, overweight, binge-eating disorder, overvaluation, psychopathology, repertory grid, internal conflict, personal construct

## Abstract

**Introduction:**

The overvaluation of weight and shape is a diagnostic criterion in eating disorders, except in binge eating disorder (BED), where it has received less attention. This aspect is also not usually analyzed in people with overweight or obesity without an eating disorder. This research aims to identify the indicators of symptomatology, as well as those of self-construction and cognitive structure, that are associated with overvaluation in obesity, either alone or in conjunction with BED.

**Method:**

A sample of 102 overweight or obese participants was accessed. The sample was divided into four groups: one without overvaluation or BED (*n* = 33); a second with overvaluation and without BED (*n* = 21); a third with BED, but without overvaluation (*n* = 15), and a fourth with BED and overvaluation (*n* = 33). The groups completed instruments regarding eating symptomatology, anxiety, depression, and stress. In addition, they were administered the Repertory Grid Technique, a semi-structured interview to evaluate the cognitive structure involved in the construal of the self and others.

**Results:**

The factors of overvaluation and the presence of BED independently explained eating symptomatology, and the latter also showed a tendency to influence anxiety, depression, and stress. In terms of cognitive structure, weight polarization was explained by overvaluation, while BED was associated with a high presence of cognitive conflicts. In self-construction, BED was the factor that explained the differences, particularly in Self-Ideal discrepancy.

**Discussion:**

The results highlight the importance of overvaluation in obesity, even in the absence of BED. Its evaluation and treatment are recommended. Furthermore, in the case of BED, it is also advisable to evaluate the overvaluation of weight and shape since it can be a severity specifier.

## 1 Introduction

Overvaluation of weight and shape is a concept that alludes to the great importance that some people attach to body weight and/or shape in assessing personal worth (Fairburn and Harrison, 2003). This is considered a central element in the development of eating disorders (EDs) (Fairburn et al., 2003). According to the DSM-5-TR, overvaluation is a diagnostic criterion for Bulimia Nervosa (BN) and Anorexia Nervosa, but not for Binge Eating Disorder (BED). However, different authors have investigated the role of overvaluation in BED, noting that this should be an element to be taken into account, either as a diagnostic criterion or as a specifier of severity in BED (e.g., Grilo, 2013; Harrison et al., 2015; Kenny and Carter, 2018; Mitchison et al., 2018; Coffino et al., 2019).

In the case of people with obesity without ED, little research has been conducted concerning the role of overvaluation of weight and shape in their mental health. Most research on the subject focuses on BED, and is based on comparisons between groups: people with obesity (without determining the presence or absence of overvaluation), people with BED, with or without overvaluation (e.g., Harrison et al., 2015; Kenny and Carter, 2018) and, in some cases, there is a group of people with BN (e.g., Grilo et al., 2010; Coffino et al., 2019). Despite the scarcity of research, there is evidence (e.g., Sonneville et al., 2015) indicating that weight overvaluation in overweight/obesity is associated prospectively with the presence of binge eating and depressive symptoms, highlighting its clinical relevance in people with obesity without ED.

When comparisons have been made between people with BED, with and without overvaluation, it has been observed that those with overvaluation generally show more symptoms of EDs, as well as worse psychological functioning (Harrison et al., 2016; Kenny and Carter, 2018), providing further evidence of the relevance of assessing this aspect. These studies were conducted predominantly in adult women, with little participation of men. In this way, one of the few studies that have been carried out with a large sample of more than a thousand treatment-seeking patients with obesity included both male and female participants without an ED. This study showed that overvaluation is associated with greater eating and general psychopathology (Dalle Grave et al., 2020). Specifically, regarding food symptomatology, it has been observed that people with BED and overvaluation present more problems in emotional regulation than those who do not overvaluate (Harrison et al., 2016). Thus, it is to be expected that such people tend to eat food to cope with unpleasant emotions, which is referred to by Ganley (1989) as emotional eating, which in turn is related to the manifestation of food craving (Verzijl et al., 2018; Dicker-Oren et al., 2022).

This study aims to analyze not only differences between people with and without weight/shape overvaluation of people with BED, as in previous research, but also explores this factor in people with overweight or obesity without BED, a less explored population. We present a secondary analysis of an investigation that compared the presence of cognitive conflicts, eating symptoms and anxious-depressive symptoms in people with obesity, with and without BED, whose first results have been previously published (Escandón-Nagel et al., 2018). To gauge the relevance of cognitive processes involved in the construal of self and others, this line of research was based on Kelly’s (1955) Personal Construct Theory. The central assumption of this theory is that each person develops a set of personal (largely implicit) hypotheses for both the self and the world, which are used for interpreting and anticipating events. Just as scientific hypotheses are made up of theoretical constructs, these are made up of personal constructs (Kelly, 1955; Botella and Feixas, 1998; Walker and Winter, 2007).

Each construct represents the grasping of a difference that a person has drawn from their experience. These distinctions are often shared with family members or friends, or incorporated from cultural narratives. For example, a person might express the construct “cares about oneself” (as opposed to “cares about others”), a distinction they use to categorize self and others. As a distinction reflecting a contrast in meaning, all personal constructs are represented by two opposite poles, and the person usually holds a preference one of the two. It is important to note that both these preferences and the constructs themselves are idiosyncratic (i.e., unique, different for each individual; see Walker and Winter, 2007). These constructs are the components of the system of meanings with which the person uniquely organizes their experience. The most common instrument used to elicit and analyze the constructs of a person is the Repertory Grid Technique (RGT; Feixas and Cornejo, 2002; Fransella et al., 2004).

One of the measures provided by the RGT is the self-ideal discrepancy (distance between current self and ideal self), which is regarded as an indicator of self-esteem because it provides information about how the person values the self on their terms. Similarly, the self-others discrepancy taps on self-perceived social isolation; and the ideal-others discrepancy alludes to the perceived adequacy of others, that is, how positive or negative is the view of others (Feixas and Cornejo, 2002). These three measures (self-esteem, perceived social isolation and adequacy of others) are important aspects of EDs and are associated with their severity (Bulik et al., 2002; Fairburn et al., 2003; Herbozo et al., 2015).

Few studies analyze the construal of the self and others in EDs. For example, Feixas et al. (2010) compared a group of women with BN with a control sample, both with an average age of 25 years, detecting a higher self-ideal discrepancy in the former. Another subsequent study with women with BN showed that they used more body-related constructs than the control sample (Dada et al., 2017).

One of the aspects of the cognitive structure (structural characteristics of the system of constructs) that can be assessed with the RGT is polarization. It reflects the extent to which the person employs extreme evaluations when judging the self and others. This measure has also been termed dichotomous interpersonal thinking and is considered to be an indicator of cognitive rigidity (García-Mieres et al., 2020). According to Alberts et al. (2012), dichotomous thinking is common in people with EDs. Another aspect of the cognitive structure refers to cognitive conflicts, distinguishing between two types: dilemmatic constructs and implicative dilemmas. The former are constructs that do not offer a clear course of action because the two poles are undesirable, so the person cannot choose one. If there are many dilemmatic constructs, the person can enter into a state of insecurity, doubt and inaction (Feixas and Saúl, 2004). On the other hand, implicative dilemmas occur when one construct in which the subject wishes to change (discrepant construct) is correlated with another in which the person is satisfied (congruent construct), so to acquire that desirable characteristic of the discrepant construct involves modifying another aspect of the congruent construct that the person wants to maintain. Thus, for some people, modifying a symptom, while desirable, may in turn mean a threat to personal identity (Feixas and Saúl, 2004). The presence of implicative dilemmas seems to distinguish, better than dilemmatic constructs, between clinical samples, such as people with depression and fibromyalgia, and control groups (Compañ et al., 2011; Feixas et al., 2014; Montesano et al., 2017). In EDs, implicative dilemmas are more prevalent in bulimia (Feixas et al., 2010) and in people with BED obesity than in those with obesity without BED (Escandón-Nagel et al., 2018). A study conducted by Dada (2014) comparing a group of people with different EDs with a control group also found that the group with EDs had more cognitive conflicts. Internal conflicts have been a recurrent topic of interest in psychology; however, they are not usually investigated, probably because there are few means of measuring them. Based on Personal Construct Theory, the RGT is offered as an instrument for this purpose (Feixas and Saúl, 2004).

Although several studies have found these measures of self-construal and cognitive structure to be associated with eating disorders, this is the first study to explore their relationship to the overvaluation of weight and shape in obesity and/or BED. Therefore, this research constitutes a novel study also because of the theoretical perspective adopted, which makes it possible to study how a person constructs their identity and the image of significant others (Feixas and Cornejo, 2002). In this way, it is possible to know the constructs a person uses to give meaning to their experience, and from this perspective, to analyze the role played by the symptoms concerning their meaning system. This focus on the vision of affected people contrasts with the bulk of research to date, which has been more focused on the researchers’ constructs and their various categorization systems, which are alien to the patients’ points of view.

Following these considerations, the objective of this study is to identify indicators of symptomatology as well as aspects of self-construction and cognitive structure that are associated with the overvaluation of weight and shape in people with overweight or obesity with and without BED. We expect the presence of overvaluation to be associated with psychopathological symptoms, discrepancies in self-construal, polarization, and presence of cognitive conflicts.

## 2 Materials and methods

### 2.1 Participants

After a non-probabilistic sampling for convenience, we divided a sample of 102 overweight and obese participants (9.8% with overweight; 90.2% with obesity) into four groups: one without overvaluation and BED (OB, *n* = 33); a second with overvaluation and without BED (OBOVE, *n* = 21); a third with BED and without overvaluation (BED, *n* = 15); and a fourth group with BED and overvaluation (BEDOVE, *n* = 33). Considering this sample size and the principle of maximum entropy (π = 1-π = 0.5), the sample obtain a maximum error of 0,097 (95% confidence level) and a theoretical value of contrast power (1-β) = 0.69.

The sample was recruited from different healthcare centers in Barcelona, as well as from notices located in public places and on social networks inviting people to participate in the study (Escandón-Nagel et al., 2018).

The inclusion criteria were to be over 18 years old with a BMI greater than or equal to 27 kg/m^2^, the criteria for pre-obesity according to the Spanish Society for the Study of Obesity (Salas-Salvadó et al., 2007). Additionally, they had to have at least primary education and an adequate understanding of Spanish. People who had undergone bariatric surgery were excluded since they tend to have greater psychopathological severity, thus constituting a group with distinctive characteristics (Sarwer et al., 2019). We also excluded people who had any medical diagnosis that could be the basis of obesity, making weight loss difficult, such as hypothyroidism or diabetes.

In the OB group, 87.9% were women, with an average age of 46.39 (*SD* = 10.85, range 21–68) and a BMI of 36.82 Kg/m^2^ (*SD* = 5.16, range 27.19–45.80); in the OBOVE group, 100% were women and the average age was 39.29 (*SD* = 12.83, range 18–66), with a BMI of 37.66 kg/m^2^ (*SD* = 7.70, range 27.47–55.84). The BED group was also mainly comprised of women (93.3%), with an average age of 41.13 (*SD* = 12.76, range 19–57) and a BMI of 38.62 kg/m^2^ (*SD* = 4.86, range 29.02–49.22). Finally, in the BEDOVE group, 90.9% were women and the average age was 38.70 (*SD* = 11.65, range 18–58), with a BMI of 38.46 kg/m^2^ (*SD* = 6.23 range 27.40–50.26). In Table 1, data on marital status, educational level and employment status are presented. Analysis using chi-square was performed to test the equivalence of the groups in these categorical variables without statistically significant differences (*p* > 0.05). However, specific values are not reported because the application condition for chi-squared was not met since the expected frequencies were less than 5. Therefore, the interpretation of the equivalence of groups in these variables must be taken with caution.

**Table 1:** Sociodemographic description of the sample: marital status, studies, employment situation.

		**Group**			
		**OB (*n* = 33)**	**OBOVE (*n* = 21)**	**BED (*n* = 15)**	**BEDOVE (*n* = 33)**
**Marital status**	Single	24.2%	19.0%	26.7%	39.4%
	Married	60.6%	66.7%	46.7%	51.5%
	Divorced/separated/widowed	15.2%	14.3%	26.7%	9.1%
**Education**	Primary education	39.4%	38.1%	26.7%	27.3%
**Level**	Vocational/Professional Training	36.4%	52.4%	53.3%	30.3%
	University studies	24.2%	9.5%	20.0%	42.4%
**Employment**	Employed	45.5%	52.4%	53.3%	39.4%
**Status**	Unemployed	15.2%	23.8%	13.13%	15.2%
	Other	39.4%	23.8%	33.3%	45.5%

OB, group without BED and without overvaluation; OBOVE, group without BED and with overvaluation; BED, group with BED without overvaluation; BEDOVE, group with BED and overvaluation.

Concerning BMI, the groups were equivalent: *F*(3,98) = 0.52; *p* = 0.673, η^2^ = 0.016; Levene test *F*(3,98) = 2.13; *p* = 0.101. In relation to age, the analyses yielded differences, with a medium effect size [*F*(3,98) = 2.75, *p* = 0.047, η^2^ = 0.078, Levene test *F*(3,98) = 3.60, *p* = 0.782], specifically between OB and BEDOVE (*p* = 0.046), the average age of the OB group being higher. For this reason, it became necessary to control this variable in subsequent analyses.

### 2.2 Instruments and measures

#### 2.2.1 Semi-structured interview for the assessment of BED

A semi-structured interview was prepared for this study, based on the diagnostic criteria for BED of DSM-5 (American Psychiatric Association, 2013), to identify the participants with this disorder.

#### 2.2.2 Eating disorder examination questionnaire

This instrument was initially developed by Fairburn and Beglin (1994) to assess eating habits and patterns over the past month. It consists of four subscales: Weight Concern, Eating Concern, Shape Concern and Restraint, together yielding an overall score. We used the Spanish version (Villarroel et al., 2011) consisting of 38 Likert scale items of 0–6 points. In our study, adequate reliability values were obtained (*α* weight concern = 0.727; *α* eating concern =0.754; *α* shape concern = 0.871; *α* restraint = 0.714; *α* total = 0.898). Two items from this instrument were utilized to assess the presence of overvaluation (“Has your weight influenced how you think about yourself as a person?” and “Has your shape influenced how you think about yourself as a person?”) These items explore whether weight and/or shape significantly impact self-perception, with a score of 5 or higher on either question indicating overvaluation. This measurement strategy has been used in several previous studies (e.g., Grilo et al., 2010; Harrison et al., 2015; Mitchison et al., 2017; Kenny and Carter, 2018). Because one of these items belongs to the Weight Concern scale and the other to Shape Concern, we corrected the scores belonging to these subscales, omitting those items. Based on the aforementioned, the following scores were utilized in this study: Overvaluation of Weight/Shape, Eating Concern, Restraint, and three scores that were adjusted due to the exclusion of two items used for the calculation of overvaluation (Corrected Weight Concern, Corrected Shape Concern and corrected overall score). Thus, the reliability of the corrected scores was α weight concern corrected = 0.641; *α* shape concern corrected = 0.856. In addition, a corrected overall score was also obtained, which yielded a Cronbach alpha of *α* total corrected = 0.884.

#### 2.2.3 Short form of the depression anxiety stress scales

This is a 21-item scale derived from the depression, anxiety and stress scale (DASS-21) (Lovibond and Lovibond, 1995). It was adapted to Spanish by Bados et al. (2005) and evaluates items on 0–3 Likert scale investigating symptoms of anxiety, depression, and stress in the previous 7 days. In this investigation, adequate reliability values were obtained (*α* depression = 0.819; *α* anxiety = 0.797; *α* stress = 0.822; *α* global = 0.902).

#### 2.2.4 The emotional eater questionnaire

This is an self-report questionnaire of 10 items on a 4-points Likert scale, developed in Spain by Garaulet et al. (2012) to evaluate emotional eating in people who are overweight or obese. It measures the influence of emotions on eating behavior. In this research, a Cronbach alpha was obtained of *α* = 0.863.

#### 2.2.5 The food craving inventory (FCI-SP)

This self-report questionnaire was created by White et al. (2002) to assess food craving by exploring how often in the previous month the respondent experienced craving regarding a list of 28 foods. For each item, the evaluated must respond on a 0–4 Likert scale. We used the Spanish version of the instrument developed by Jáuregui et al. (2010) which yielded a Cronbach alpha of *α* = 0.881.

#### 2.2.6 The repertory grid technique

The RGT is a semi-structured interview designed to evaluate the system of constructs of the person from which a matrix with three components is obtained. In the columns, at the top, elements (“present self,” “ideal self” and significant others of the evaluated person, such as partner and friends) are recorded (in this study, from 10 to 20 elements, depending on the participant). In the rows, the constructs provided by the person are annotated, having been elicited from the comparison, in terms of similarities and differences, between these elements taken in rotative pairs. The number of constructs elicited with this procedure varied across participants, ranging from 10 to 37. In the cells, formed by the intersection between elements and constructs, participants are asked to score each element in each construct, using a 7-point Likert scale. The whole process lasted between 50 and 70 min depending on the participant (see these manuals for more detail: Feixas and Cornejo, 2002; Fransella et al., 2004).

With this instrument, the self-construction indices were obtained, such as the self-ideal discrepancy (Euclidian distance between the scores of the “current self” and those of the “ideal self”), self-others discrepancy (Euclidian distance between the scores of the “current self” and the average of the scores attributed to others), and others-ideal discrepancy (Euclidian distance between the scores of the average of the scores attributed to others and those of the “ideal self”). In each of them, the higher the score obtained, the greater the discrepancy. It was decided to include “fat-thin” as a provided construct. This allowed for an evaluation of the perception of the current weight and the ideal weight according to the score rated for the current “self” element (1 very fat; 7 very thin). The difference between the score on the current self and the ideal self in this construct was considered a measure of body dissatisfaction. In addition, cognitive structure indices such as total polarization, polarization on the “fat-thin” construct, percentage of implicative dilemmas, and percentage of dilemmatic constructs were obtained. The higher the scores obtained in these indices, the higher the level at which the target characteristic is presented.

#### 2.2.7 Sociodemographic questionnaire

A sociodemographic questionnaire was also used to collect data to describe the sample in terms of age, sex, educational level, employment situation and marital status while collecting information on weight and size, to obtain BMI.

### 2.3 Procedure

The research was approved by the Bioethics Commission of the *Universitat de Barcelona*. We invited different health centers in Barcelona to participate in the study. Professionals of these centers informed potential participants and, if they agreed, contacted the coordinator of the study (first author). We also used posters on public sites and on social networks to invite more participants. Potential participants were informed about the study and a signature of informed consent was requested, guaranteeing the confidentiality of the data, to subsequently administer the different instruments listed above.

The psychological evaluation was carried out individually in a single session of approximately 2 h. First, the evaluator administered the sociodemographic questionnaire and conducted the semi-structured interview to assess BED. Next, the other questionnaires were applied, and finally, the RGT was administered.

After inclusion in the study, the four groups mentioned above were formed based on the information collected with the semi-structured interview to evaluate BED and on the two items of the eating disorder examination questionnaire (EDE-Q) with which overvaluation was measured.

### 2.4 Data analysis

ANCOVA was used to compare the symptomatology between the different groups, considering two factors: the presence of overvaluation and the presence of BED. Age was included as a covariate since the groups were not equivalent in this regard. Homoscedasticity was evaluated with Levene’s test. When the assumption of homoscedasticity was not met, the nonparametric statistic of Kruskall Wallis was used and, if significant values were obtained, Mann–Whitney’s *U* was used as a *post hoc*.

Self-construction and cognitive structure were compared using MANCOVA. If Box M confirmed homoscedasticity compliance for multivariate analyses, Wilks’ Lambda was used. If not, we used Pillai’s trace to interpret the results.

As for statistical programs, for the analysis of grid data, we used GRIDCOR 6.0 (Garcia-Gutierrez and Feixas, 2018) and results of the variables from RGT were introduced to the overall analysis of the data that was performed with IBM SPSS 22. The value of the type I error or global alpha risk was set to α < 0.05; however, when multiple comparisons were used in ANCOVA, MANOVA and Kruskall Wallis post-hoc, we applied the Bonferroni correction for groups of variables (the corresponding corrected value is indicated in the Results section). In addition to the *p*-value, we reported the effect size of the different analyses.

## 3 Results

### 3.1 Symptoms of depression, anxiety, stress, and eating behavior

In Table 2, descriptive statistics obtained by the different subgroups in the symptomatology of depression, anxiety, and stress, as well as in symptoms of food behavior are presented.

**Table 2:** Descriptive statistics of symptoms of depression, anxiety, stress and eating behavior.

	**With overvaluation**		**Without overvaluation**	
	**Presence of BED**		**Presence of BED**	
**Variable**	**Yes (*n* = 33) *M(SD)***	**No (*n* = 21) *M(SD)***	**Yes (*n* = 15) *M(SD)***	**No (*n* = 33) *M(SD)***
DASS-21 depression	20.61 (12.95)	14.76 (13.09)	16.93 (8.81)	12.73 (10.80)
DASS-21 anxiety	15.09 (8.90)	13.52 (11.04)	15.73 (9.47)	8.42 (9.27)
DASS-21 stress	20.18 (9.64)	20.00 (8.34)	22.13 (7.39)	15.15 (11.10)
EDE-Q corrected total	3.75 (0.93)	3.33 (0.66)	3.03 (0.93)	2.06 (1.02)
EDE-Q restriction	2.49 (1.53)	2.64 (1.23)	1.69 (1.43)	2.06 (1.42)
EDE-Q corrected weight	4.13 (1.06)	3.92 (1.07)	3.37 (1.31)	2.27 (1.16)
EDE-Q corrected shape	5.04 (0.82)	4.73 (0.81)	4.29 (1.38)	2.89 (1.47)
EDE-Q eating concern	3.35 (1.46)	2.02 (1.07)	2.76 (0.91)	1.00 (1.11)
EEQ	22.33 (4.13)	15.00 (3.87)	19.73 (4.48)	10.70 (5.58)
FCI-SP	44.45 (15.25)	33.76 (9.49)	43.27 (12.74)	27.27 (19.28)
Body dissatisfaction	3.94 (1.48)	3.90 (1.61)	3.13 (1.41)	3.33 (1.57)

BED, Binge Eating Disorder; DASS-21, Short form of the Depression Anxiety Stress Scales; EDE-Q, Eating Disorder Examination Questionnaire; EEQ, Emotional Eater Questionnaire; FCI-SP, Food Craving Inventory.

Regarding ANCOVA, Table 3 emphasizes that, considering the correction of Bonferroni (*α* < 0.013), no differences in depression, anxiety, or stress were detected.

**Table 3:** Comparative analysis of symptoms of depression, anxiety, and stress.

**Variable**	**Comparison**	***F***	**d.f.**	***p***	**η** ^**2**^
DASS-21	Overvaluation	1.37	1, 97	0.203	0.017
Depression	Presence of BED	4.46	1, 97	0.037*	0.044
	Overvaluation × BED	0.07	1, 97	0.797	0.001
	Age	0.59	1, 97	0.446	0.006
	Levene’s test	1.26	3, 98	0.294	
DASS-21	Overvaluation	1.36	1, 97	0.246	0.014
Anxiety	Presence of BED	5.02	1, 97	0.027*	0.049
	Overvaluation × BED	2.14	1, 97	0.147	0.022
	Age	0.17	1, 97	0.679	0.002
	Levene’s test	0.74	3, 98	0.532	
DASS-21	Overvaluation	0.85	1, 97	0.360	0.009
Stress	Presence of BED	3.62	1, 97	0.060	0.036
	Overvaluation × BED	3.21	1, 97	0.077	0.032
	Age	1.24	1, 97	0.269	0.013
	Levene’s test	1.40	3, 98	0.248	

*Significant to the level 0.05.

As shown in Table 4, concerning the corrected total EDE-Q, differences in scores explained by both factors were independently detected. That is, on the one hand, people with overvaluation had higher scores than those who did not overvalue weight and shape (with a large effect size), and those with BED scored higher than those who did not (with a medium effect size). Something similar happened with Corrected Weight Concern and Eating Concern, as in both cases, the scores were explained by the two factors independently.

**Table 4:** Comparative analysis of symptoms of eating behavior.

**Variable**	**Comparison**	***F***	**d.f.**	***p***	**η** ^**2**^
**EDE-Q**	Overvaluation	25.91	1, 97	<0.001**	0.211
**Total**	Presence of BED	12.90	1, 97	<0.001**	0.117
**Corrected**	Overvaluation × BED	1.96	1, 97	0.165	0.020
	Age	0.00	1, 97	0.955	0.000
	Levene’s test	0.95	3, 98	0.421	
**EDE-Q**	Overvaluation	6.00	1, 97	0.016*	0.058
**Restriction**	Presence of BED	0.58	1, 97	0.450	0.006
	Overvaluation × BED	0.85	1, 97	0.771	0.001
	Age	0.84	1, 97	0.361	0.009
	Levene’s test	0.70	3, 98	0.557	
**EDE-Q**	Overvaluation	22.94	1, 97	<0.001**	0.191
**Corrected weight**	Presence of BED	6.84	1, 97	0.010**	0.066
**Concern**	Overvaluation × BED	3.08	1, 97	0.082	0.031
	Age	1.07	1, 97	0.303	0.011
	Levene’s test	0.56	3, 98	0.643	
**EDE-Q**	Overvaluation	9.18	1, 97	0.003**	0.086
**Eating**	Presence of BED	36.16	1, 97	<0.001**	0.272
**Concern**	Overvaluation × BED	0.63	1, 97	0.429	0.006
	Age	0.25	1, 97	0.615	0.003
	Levene’s test	2.57	3, 98	0.059	
**EEQ**	Overvaluation	13.52	1, 97	<0.001**	0.122
	Presence of BED	71.84	1, 97	<0.001**	0.425
	Overvaluation × BED	0.94	1, 97	0.335	0.010
	Age	1.03	1, 97	0.312	0.011
	Levene’s test	2.45	3, 98	0.068	
**Body dissatisfaction**	Overvaluation	4.72	1, 97	0.032*	0.046
	Presence of BED	0.05	1, 97	0.830	0.000
	Overvaluation × BED	0.11	1, 97	0.741	0.001
	Age	0.12	1, 97	0.733	0.001
	Levene’s test	0.80	3, 98	0.497	

*Significant to the level 0.05. **Significant to the level 0.013.

A trend (without reaching statistical significance according to the corrected alpha value, but with a value of less than *p* < 0.05) can be seen in Table 4 for the Restriction scale relative to the weight and shape overvaluation factor, regardless of BED. With a medium effect size, the group’s scores with overvaluation were higher. Something similar happened with body dissatisfaction, although in this case, the magnitude of the difference was small.

Regarding the emotional eater questionnaire (EEQ), differences were identified between the groups given by both factors independently, with higher scores on those with BED, as well as in the overvaluation group. As shown in Tables 3, 4, in none of the analyses did the age covariate turn out to be statistically significant.

In the variables Corrected Shape Concern [*F*(3,98) = 6.17; *p* = 0.001] and Food Craving [*F*(3,98) = 2.91; *p* = 0.038] the homoscedasticity condition was not met and the Kruskal Wallis analysis was used. For Mann–Whitney’s *U post-hoc* analyses, the corrected alpha was 0.008. In Corrected Shape Concern, differences were found between the groups [χ^2^(3) = 36.7, *p* < 0.001]. The post-hoc analyses are presented in Table 5, which shows that specifically there are differences, with a large effect size, between the OB group and BEDOVE; between the OB and OBSOB group, with a medium effect size; and between OB and BED, with a small effect size. Differences were also identified in the Food Craving variable between the groups [χ^2^ (3) = 21.50, *p* < 0.001]. As shown in Table 5, the main differences were between OB and BED and OB and OBOVE, in both cases with a medium effect size.

**Table 5:** Nonparametric group comparison analysis in EDE-Q corrected shape concern and FCI-SP.

**Variable**	**Groups**		***U***	***Z***	***p***	***r***
EDE-Q corrected shape concern	OB	BED	116.50	−2.92	0.004**	0.29
		BEDOVE	117.00	−5.49	<0.001**	0.54
		OBOVE	95.50	−4.46	<0.001**	0.44
	BED	BEDOVE	165.5	−1.83	0.067	0.18
		OBOVE	130.00	−0.89	0.375	0.09
	BEDOVE	OBOVE	283.00	−1.13	0.257	0.11
FCI-SP	OB	BED	107.00	−3.13	0.002**	0.31
		BEDOVE	245.00	−3.84	<0.001**	0.38
		OBOVE	244.00	−1.82	0.069	0.18
	BED	BEDOVE	242.00	−0.12	0.903	0.01
		OBOVE	82.00	−2.43	0.015*	0.24
	BEDOVE	OBOVE	198.00	−2.64	0.008**	0.26

OB, group without BED and without overvaluation; OBOVE, group without BED and with overvaluation; BED, group with BED without overvaluation; BEDOVE, group with BED and overvaluation. *Significant to the level 0.05; **Significant to the level 0.008.

### 3.2 Self-construction and cognitive structure

Table 6 presents the descriptive statistics referring to self-construction and cognitive structure.

**Table 6:** Descriptive statistics for measures of self-construction and cognitive structure.

	**With overvaluation**		**Without overvaluation**	
	**Presence of BED**		**Presence of BED**	
	**Yes (*n* = 33)**	**No (*n* = 21)**	**Yes (*n* = 15)**	**No (*n* = 33)**
	***M(SD)***	***M(SD)***	***M(SD)***	***M(SD)***
Self-ideal discrepancy	0.39 (0.11)	0.31 (0.11)	0.35 (0.10)	0.28 (0.10)
Self perceived social isolation	0.29 (0.07)	0.26 (0.06)	0.28 (0.06)	0.25 (0.06)
Perceived adequacy in others	0.25 (0.07)	0.23 (0.06)	0.24 (0.06)	0.22 (0.05)
Actual weight perception	1.33 (0.60)	1.61 (0.80)	1.53 (0.83)	1.79 (0.78)
Ideal weight perception	5.27 (1.28)	5.52 (1.25)	4.67 (1.40)	5.12 (1.08)
Total polarization	29.35 (12.72)	35.05 (16.09)	33.41 (16.72)	34.44 (19.30)
Weight polarization	30.83 (18.07)	27.48 (18.97)	20.34 (12.00)	17.30 (15.35)
Implicative dilemmas	11.25 (15.08)	7.94 (17.98)	22.81 (29.83)	5.51 (10.11)
Dilemmatic constructs	22.28 (16.32)	15.39 (17.55)	13.04 (12.23)	13.73 (11.55)

Concerning the self-construction variables, to compare the samples using MANCOVA, the homogeneity of the variance–covariance matrixes was checked with the Box’s *M* test, confirming compliance with this assumption [*F*(45,11454.02) = 1.00, *p* = 0.468]. In addition, as shown in Table 7, the Levene test found homoscedasticity in all the variables involved. The source of variation presence of BED was statistically significant for the explanation of the variability of self-construction [*F*(5,93) = 3.74, *p* = 0.004; Wilks’ Lambda = 0.833; η^2^ = 0.167], with a large effect size. This is explained, specifically, by the self-ideal discrepancy (Table 7), which was higher in the BED group, regardless of overvaluation. The other sources of variation do not explain the variability of self-construction [Overvaluation *F*(5,93) = 1.03, *p* = 0.402; Wilks’ Lambda = 0.947; η^2^ = 0.053; the presence of BED × Overvaluation (*F*(5,93) = 0.07, *p* = 0.996; Wilks’ Lambda = 0.996; η^2^ = 0.004]. In addition, the age covariate also did not prove statistically significant [*F*(5,93) = 0.38, *p* = 0.863; Wilks’ Lambda = 0.980; η^2^ = 0.020].

**Table 7:** Comparative analysis of self-construction.

**Variable**	**Comparison**	***F***	**d.f**	***p***	**η** ^**2**^
Discrepancy ideal-self	Overvaluation	1.46	1, 97	0.230	0.015
	Presence of BED	10.52	1, 97	0.002**	0.098
	Overvaluation × BED	0.01	1, 97	0.911	0.000
	Age	0.73	1, 97	0.394	0.008
	Levene’s test	0.08	3, 98	0.969	
Self perceived social isolation	Overvaluation	0.31	1, 97	0.581	0.003
	Presence of BED	3.89	1, 97	0.051	0.039
	Overvaluation × BED	0.03	1, 97	0.860	0.000
	Age	0.47	1, 97	0.494	0.004
	Levene’s test	0.30	3, 98	0.826	
Perceived adequacy in others	Overvaluation	1.25	1, 97	0.267	0.013
	Presence of BED	1.75	1, 97	0.189	0.018
	Sobrevaloración × BED	0.16	1, 97	0.686	0.002
	Age	0.00	1, 97	0.999	0.000
	Levene’s test	0.34	3, 98	0.796	
Actual weight perception	Overvaluation	1.70	1, 97	0.195	0.017
	Presence of BED	3.30	1, 97	0.073	0.033
	Overvaluation × BED	0.00	1, 97	0.974	0.000
	Age	0.51	1, 97	0.478	0.005
	Levene’s test	1.22	3, 98	0.307	
Ideal weight perception	Overvaluation	3.66	1, 97	0.059	0.036
	Presence of BED	1.83	1, 97	0.179	0.019
	Overvaluation × BED	0.15	1, 97	0.696	0.002
	Age	0.00	1, 97	0.998	0.000
	Levene’s test	1.14	3, 98	0.336	

*Significant to the level.05. **Significant to the level 0.013.

Regarding the cognitive structure, Box’s *M* test indicates that the homogeneity of the matrixes of variance–covariance is not met [*F*(30, 12491.41) = 2.17, *p* < 0.001]. Nevertheless, with the Levene test, the homoscedasticity was stated in almost all the implied variables, as seen in Table 8, except in the percentage of implicative dilemmas [*F*(3,98) = 4.35; *p* = 0.006].

**Table 8:** Comparative analysis of cognitive structure.

**Variable**	**Comparison**	**F**	**d.f**	**p**	**η** ^**2**^
Polarization	Overvaluation	0.00	1, 97	0.999	0.000
	Presence of BED	1.30	1, 97	0.257	0.013
	Overvaluation × BED	1.16	1, 97	0.285	0.012
	Age	1.17	1, 97	0.283	0.012
	Levene’s test	1.90	3, 98	0.135	
Weight polarization	Overvaluation	8.87	1, 97	0.004**	0.084
	Presence of BED	0.91	1, 97	0.343	0.009
	Overvaluation × BED	0.00	1, 97	0.996	0.000
	Age	0.18	1, 97	0.676	0.002
	Levene’s test	2.00	3, 98	0.119	
Percentage of dilemmatic constructs	Overvaluation	2.24	1, 97	0.138	0.023
	Presence of BED	0.73	1, 97	0.396	0.007
	Overvaluation × BED	1.86	1, 97	0.176	0.011
	Age	1.80	1, 97	0.183	0.018
	Levine’s test	1.26	3, 98	0.292	

*Significant at the level 0.05. **Significant at the level 0.013.

The source of overvaluation variation is statistically significant for the explanation of the variability of cognitive structure [*F*(4,94) = 4.03, *p* = 0.005; Pillai trace = 0.146; η^2^ = 0.146], with a large effect size. Specifically, as shown in Table 7, the weight polarization is more marked in the group with overvaluation. In addition, the presence of BED also explains the variability of cognitive structure [*F*(4,94) = 3.63, *p* = 0.009; Pillai trace = 0.134; η^2^ = 0.134], with a large effect size.

The interaction of BED and overvaluation does not explain the variability in cognitive structure [*F*(4,94) = 1.62, *p* = 0.176; Pillai trace = 0.064; η^2^ = 0.064]. The age covariate also did not prove statistically significant [*F*(4,94) = 0.82, *p* = 0.516; Pillai trace = 0.034; η^2^ = 0.034].

In implicative dilemmas, Kruskal Wallis was used to identify differences between the groups [χ^2^ (3) = 9.45, *p* = 0.024]. Although, as shown in Table 9, Mann–Whitney’s *U post-hoc*-based analyses did not yield statistically significant differences when considering the corrected alpha value of 0.008.

**Table 9:** Nonparametric group comparison analysis in implicative dilemmas.

**Variable**	**Groups**		***U***	***Z***	***p***	***r***
Implicative dilemmas	OB	BED	143.50	−2.55	0.011*	0.252
		BEDOVE	395.00	−2.07	0.038*	0.204
		OBOVE	335.50	−0.23	0.819	0.023
	BED	BEDOVE	197.5	−1.14	0.253	0.113
		OBOVE	96.50	−2.09	0.036*	0.207
	BEDOVE	OBOVE	260.50	−1.62	0.105	0.160

OB, group without BED and without overvaluation; OBOVE, group without BED and with overvaluation; BED, group with BED without overvaluation; BEDOVE, group with BED and overvaluation. *Significant at the level 0.05; **Significant at the level 0.008.

## 4 Discussion

This study sought to identify which indicators of symptomatology, self-construction, and cognitive structure, are associated with overvaluation of weight and shape in people with obesity (or overweight), either alone or in conjunction with BED. The main results indicate that global eating symptomatology, as well as specific concerns about weight, eating and emotional eating, are explained by overvaluation and by BED independently. On the one hand, this confirms previous findings regarding the increased psychopathological severity of people with BED and overvaluation, compared to those with BED but who do not exhibit overvaluation (Harrison et al., 2016; Kenny and Carter, 2018). In addition, this study has provided evidence of the importance of assessing the overvaluation of weight and shape in people with obesity without BED since even in these cases, it is associated with a risk of experiencing symptoms of EDs (Sonneville et al., 2015).

In addition, we found a trend regarding food restriction which appeared to be higher in the groups that overvalued weight and shape, regardless of the presence of BED. This is important since, as Fairburn et al. (2003) pointed out, restriction is a relevant factor in the emergence of binge eating, and being part of the core psychopathology of EDs. In other words, restriction is associated with EDs, regardless of the specific diagnosis, and is also associated with overvaluation of weight and shape, even in obese patients without eating disorders. At this point, it is important to highlight that although restriction is not a diagnostic criterion for BED, and is even an aspect to consider in the differential diagnosis with BN, there is evidence that people with BED engage in constant attempts at dieting, behavior based on food restriction. In a recent study based on interviews with experts, a consensus was reached that food restriction is a common behavior in BED, whether voluntary or imposed by a third party (such as a health professional) (Bray et al., 2023).

Regarding emotional eating and food craving, these variables were higher in those who overvalued weight and shape, as well as in those who presented BED, which coincides with previous findings (Harrison et al., 2016; Verzijl et al., 2018), thus corroborating the importance of considering these variables in the presence of overvaluation.

As for the symptomatology of anxiety, depression, and stress, they are not explained by overvaluation, although a trend in depression and anxiety regarding the presence of BED is detected, being greater in those with the eating disorder, as found by Klatzkin et al. (2015).

On the other hand, in the cognitive structure, the polarization of weight was explained by overvaluation and not by BED. This implies that people with obesity or overweight and who overestimate the importance of their weight and shape have a more dichotomous way of thinking concerning their weight and that of others compared to those who do not overestimate. So, for such people, there are only two extreme positions: being “fat” or “thin.” Therefore, it can be hypothesized that when they are in the “fat” pole, the “thin” pole is perceived as distant, an unattainable goal for them. This result goes in line with the contributions of Alberts et al. (2012) pointing out that dichotomous thinking is important in EDs. However, the findings of this study indicate that in the case of people with obesity (or overweight), dichotomous thinking is important only with a particular type of content of thought, the weight, which is linked to the presence of an overestimation of the importance of weight and shape. This finding is aligned with the contributions of Palascha et al. (2015) indicating that dichotomous thinking about food and diet is associated with diet stiffness behaviors, making it difficult to maintain adequate body weight.

The results of this study suggest that cognitive structure in obesity is also explained by the presence of BED, but in this case, it seems to be the implicative dilemmas that take on importance since they occur in greater numbers in those who have BED, regardless of overvaluation. That is, those who have BED tend to present more conflicted cognitive systems, which coincides with what is observed in samples with different psychopathological profiles (Montesano et al., 2015; Escandón-Nagel et al., 2018). However, more studies are needed to confirm this finding regarding BED.

As for the construction of self, it was also the presence of BED that best explained the differences. Specifically, the variable self-ideal discrepancy was higher in those who presented BED than those who did not present BED, regardless of overvaluation. This indicates that those who have obesity and BED show more damaged self-esteem than those who have only obesity, which is in line with previous literature (Bulik et al., 2002; Herbozo et al., 2015).

The measures employed in this study, based on Personal Construct Theory, have allowed the recognition of important aspects of personal identity, which have distinguished individuals with obesity with and without BED. Regarding weight polarization, it complements the existing literature on polarized or dichotomous thinking in EDs, recognizing the importance of dichotomous thinking regarding one’s own weight and/or that of others.

The main limitation of this study is the difference in the sample size of the subgroups since the BED and OBOVE groups are smaller in comparison to the rest. In addition, there is generally a low representation of men in the sample.

The methodological limitations of this study should be taken into account when analyzing the results, since it is a cross-sectional study that, by its nature, does not allow establishing causal relationships. This implies that it is not possible to determine from these data whether the overvaluation of weight and shape is at the basis of the symptomatology studied or whether it is a consequence of it.

For future research, it would be important to analyze whether these findings apply to men with overweight or obesity. Also, considering that BED can occur in people with normal weight, it would be interesting to investigate the role of overvaluation of weight and shape in such cases.

Previous research has shown that overvaluation of weight and shape acts as a moderator of the relationship between self-esteem and internalization of weight stigma in people with obesity and BED (Pearl et al., 2014). This relationship is associated with negative health outcomes, such as a significant presence of obesogenic behaviors (Puhl and Lessard, 2020). Therefore, it would be interesting for future research to analyze in depth the role of overvaluation in terms of weight stigma, not only in people with obesity and binge eating but also in those who do not present BED.

The results of this study also provide evidence of the overlap between obesity and eating disorders. Although they are different phenomena, the evidence shows that there are some elements that are common to both. As observed in the present study, there is a subgroup of individuals with obesity who also exhibit an overvaluation of weight and shape. Along the same lines, research such as that of Breton et al. (2022) emphasizes that an interdisciplinary approach is essential to achieve a better understanding of these phenomena and the elements that are common to both.

We expected that the presence of overvaluation of weight and shape would be associated with more psychopathological symptoms, as well higher discrepancies in self-construction, higher polarization, and a higher percentage of implicative dilemmas and dilemmatic constructs. This hypothesis was partially confirmed, since the overvaluation was indeed associated with eating psychopathology, but not with anxiety, depression, or stress. Regarding cognitive structure, overvaluation was associated with a greater polarization of weight, while self-construction did not explain the presence of overvaluation, although it was linked to the presence of BED, as it was found for other EDs (e.g., Feixas et al., 2010; Dada, 2014).

The results of this study highlight the importance of weight and shape overvaluation in the evaluation and treatment not only of people with BED but also of those with overweight-obesity without an ED. The interest in this variable is justified not only by its influence on symptomatology but also by the cognitive structure that underpins personal identity.

This study recognizes the importance of weight polarization, dietary restriction, and emotional eating, concerning overvaluation. In future research, it would be relevant, then, to investigate the role that these variables have regarding the development of an ED such as BED.

All this provides possible focuses of action in terms of intervention, suggesting investigation and targeting of the overvaluation of weight and shape be conducted as early as possible, particularly in patients with obesity or overweight who do not manifest a diagnosis of BED.

## 5 Conclusion

Overvaluation of weight and shape is considered an important part of EDs like anorexia and bulimia, but not of BED. Also, there has been little research on what role this variable plays in people with overweight or obesity. In our sample of people with overweight or obesity we studied the role of the presence of BED and/or overvaluation, finding that the latter was associated with higher eating symptomatology not only in people with BED but also in obesity in general. Overvaluation of weight and shape seems to be a factor related to the severity of BED. In addition, overvaluation in people with obesity or overweight (not the presence of BED) was associated with higher polarization of ratings of weight for the self and others. For future research, it would be interesting to evaluate whether, in the long term, people with obesity and without BED, who at some point present overvaluation of weight and shape, will eventually develop an ED.

## Data availability statement

The raw data supporting the conclusions of this article will be made available by the authors, without undue reservation.

## Ethics statement

The studies involving humans were approved by The Bioethics Commission of the University of Barcelona (IRB00003099). The studies were conducted in accordance with the local legislation and institutional requirements. The participants provided their written informed consent to participate in this study.

## Author contributions

NE-N: Writing – original draft, Project administration, Methodology, Formal analysis, Conceptualization. MP: Writing – review & editing, Project administration, Methodology, Formal analysis. AG: Writing – review & editing, Resources, Investigation. JS: Writing – review & editing, Resources, Investigation. GF: Writing – review & editing, Project administration, Methodology, Conceptualization.
